# The effects of social concern goals on the value of learning and on the intentions of medical students to change their majors

**DOI:** 10.1080/10872981.2017.1330631

**Published:** 2017-06-05

**Authors:** Soowon Park, Seunghee Cho, Jun-Young Lee

**Affiliations:** ^a^ Department of Education, Sejong University, Seoul, Republic of Korea; ^b^ Woodside Priory School, Portola Valley, CA, USA; ^c^ Department of Psychiatry, Seoul National University & SMG-SNU Boramae Medical Center, Seoul, Republic of Korea

**Keywords:** Goal content, social concern goal, medical students

## Abstract

**Background**: In the process of developing a professional medical expertise, goals can become a psychological impetus and act as a source of retaining an individual’s persistency. Therefore, the goals of medical students should be considered when designing a curriculum for health professions.

**Purpose**: The purpose of this study was to examine relative effects of goal categories on the value of learning and intention to change one’s major.

**Method**: Data were obtained from the Korea Education Longitudinal Study, which included 1938 representative Korean college freshmen majoring in medicine, engineering, natural science and humanities. They answered a survey questionnaire about goal categories (i.e., social concern, affiliation, self-growth, leisure, wealth, and fame), the value of learning, and intention to change one's major.

**Results**: For medical students, social concern goals were positively related to the value of learning and negatively related to the intention to change one's major. Social concern goals decreased the intention to change one's major directly, and also indirectly through increased value of learning.

**Conclusion**: Providing context for enhancing medical students’ social concern goals is necessary in a medical training curriculum, not only for the students’ professional development but also for improving society.

**Abbreviations**: GCT: Goal contents theory GPA: Grade point average KELS: Korea education longitudinal study SDLA: Self-directed learning abilities SDT: Self-determination theory

## Introduction

Most researchers and educators agree that social concern is fundamental in medical professionals. Social concern motivation is not only an important prerequisite for developing medical students’ expertise, but also for the desired outcome of medical education. Therefore, there is a vital interest among researchers in understanding what influences and what results from students’ social concern desire. The purpose of this study was to identify medical students’ goal contents and their effects on their college life (i.e., value of learning and intention to change one's major), especially focusing on their social concern goals by comparing medical students’ goal contents with those of other majors (i.e., engineering, natural science, and humanities).

Students strive to live a successful life in which they feel they have reached their dreams and aspirations. In order to accomplish such a life, they tend to have specific goals in mind to help guide them throughout their lives. Goals are inner representations of desired results and are used as motivation for individual behavior. Goals are directly related to behavior in that they can increase motivation levels for tasks, control effort levels, and overcome failures in the process of intensive training to reach a certain level of expertise [1]. However, the goals of medical students are relatively unacknowledged and overlooked. Thus, medical teachers should be more aware of the goals of medical students and help them pursue more adaptive goals in their careers and professional development.

According to goal contents theory (GCT), which is a mini theory of self-determination theory (SDT), consequences differ depending on whether the goals are intrinsic or extrinsic. Intrinsic goals are inwardly oriented aspirations or personal inner growth, which reflect authentic motives. Social concern, helping others, and relationships are examples of intrinsic goals. Extrinsic goals are outwardly-oriented aspirations and are deeply related to compensation or obtaining approval of others. Examples of extrinsic goals are economic success, social recognition, and appearance [2].

Intrinsic goals are associated with being more optimistic about personal psychological well-being and motivation compared to extrinsic goals [3,4]. Adolescents who pursue intrinsic goals tend to have better mental health, academic performance, and self-efficacy and to demonstrate better adaptive skills. Conversely, adolescents pursuing extrinsic goals tend to feel more depressed and anxious, and to demonstrate behavioral disorders compared to those who pursue intrinsic goals. Many current studies commonly report that extrinsic goals have negative effects [5].

Since an individual pursues multiple goals at the same time, relative effects of different goal categories should be considered. Social concern is one of the representative intrinsic goals that is associated with the desire to contribute to society. This goal content reflects an altruistic nature and is a beyond-the-self societal contribution. In line with the ‘social responsibility and concern’ [6] of social goals, a motivation to contribute toward the betterment of society and a concern for others’ welfare are basic motivations for this goal. Recent studies revealed that a self-transcendent purpose can foster academic self-regulation [7] and that pursuing a social concern goal positively predicts behavioral, emotional, and cognitive academic engagement in school (i.e., elaboration, monitoring, and regulation) after controlling for the mastery and performance goal orientation [8]. Pursuing a social concern goal in academic situations facilitates perspectives that a task is meaningful [8,9]. Observational field studies revealed that when people who have jobs with low status that also require extremely repetitive work such as trash collecting, focus on the benefit of others; they consider their role meaningful and are able to work more efficiently [10,11]. These studies showed that pursuing a social concern goal could impact positively on students’ motivation.

Since altruism is a critical factor for treating people’s illnesses, it has long been studied in medical students [9,10]. However, there is relatively low interest in the effects of social concern motives or cultivation of altruism on medical students’ emotional or academic aspects. If the social concern pursuit has a detrimental effect on students’ motivation, encouraging them to have social concern goals should be under reconsideration. Thus, an empirical study for investigating the consequences of social concern motivation is critical; however, this area remains largely unacknowledged in medical education.

Affiliation, self-growth, leisure, wealth, and fame are other goal categories that students could pursue. Affiliation is an intrinsic goal, a yearning for emotional ties with others (e.g., lovers and friends). People naturally want to belong to a group and be a part of a group as a social being. According to Erikson’s stages of development, psychological strives for intimacy are heightened in the early 20s [8]. Thus, affiliation is a goal that motivates students. Self-growth is also an important goal content in individuals’ learning and professional development. A self-growth goal reflects aspirations for growth and focuses on one’s development. People with expertise showed higher self-growth goals than non-experts, and self-growth goals are considered a prerequisite for developing expertise [11]. Thus the self-growth goal is important for students who are on their expertise-developing paths. A leisure goal indicates valuing resting time away from education, work, or chores. Balancing life between work and leisure is important in today’s society. Therefore, a leisure goal is also to be pursued. Wealth is an extrinsic goal which motivates a person by materialistic compensations and financial success [5]. Many adolescents tend to want stable jobs that can not only sustain their lives but also allow them to consume products other than necessities. Thus, wealth could be a major motivation for college students. Lastly, fame is an extrinsic goal associated with wishing to be acknowledged, famous and admired by others. Fame, alongside wealth, could also be an indicator that can measure success in society; thus, it could also be a motivation [12].

In this research we looked into how goal contents are related to learning motivation (i.e., value of learning and intention to change major) for medical students. Based on the perspectives of GCT, academic motivation can be increased depending on which goal content students choose to focus on. The specific goal categories we focused on in this study are social concern, affiliation, self-growth, leisure, wealth, and fame. Among the goal categories, social concern is to be especially noted in this study. The authors also examined how intrinsic goals, in comparison to extrinsic goals, are associated with motivation to learn in medical students. In the educational context, educators should be able to help students formulate more adaptive goals that help them take more interest in learning and help them be more willing to continue with their major. With this study, hopefully students will gain perspectives on how to construct their goal contents in order to achieve a more adaptive college life. The present study addressed the following research questions:

1. How were goal contents associated with value placed on learning in each major group?
2. How were goal contents associated with intention to change one's major in each major group?
3. How were goal contents, value placed on learning, and intention to change one's major associated in each major?

## Methods

### Participants

This study used the panel data from the Korea Education Longitudinal Study (KELS). This national survey was launched in 2005 when students were in their first year of middle school. A follow-up survey was conducted each subsequent year. The data were collected using a stratified cluster random sampling, and the sample of KELS is representative of Korean adolescents. Written consent was obtained from all participants through their school. The present study utilized data at time 7 (2011), when participants became first-year college students. The total number of participants in 2005 was 6825 (3565 males and 3260 females), but in the seven years of follow-up 1975 (28.93%) were dropped, thus a total of 4850 (2464 male and 2386 female) participants remained. Among them, 3582 participants were currently registered in college. In this study, a total of 1938 college students who were majoring in medicine (318; 71 males), engineering (852; 663 males), natural science (418; 203 males), and humanities (350; 119 males) were included for data analysis.

### Measures

Questions about students’ demographic factors (major, gender, etc.), goal contents, value placed on learning, intention to change one's major, study time per week, grade point average (GPA), perceived self-directed learning abilities (SDLA), educational satisfaction and relational satisfaction were used. Values placed on learning and intentions to change one's major were used as dependent variables. Gender, study time per week, GPA, SDLA, educational satisfaction, and relational satisfaction were used as control variables.

### Goal contents

Designed to measure various types of goals, six goal categories (i.e., wealth, fame, social concern, affiliation, self-growth, and leisure) were adopted from the KELS. Each goal category was measured by four items. Examples of items were ‘It is important to give back to society what I gained from society (social concern)’, ‘I consider it important to form good relationships with other people (affiliation)’, ‘Continuing one’s effort to develop an individual’s potential is important in life (self-growth)’, ‘I consider leisure more important than work or study (leisure)’, ‘When making a career choice, annual income is more important than other factors (wealth)’, ‘I consider it important to be an authoritative figure in my field of expertise (fame)’. For each question, a five-point Likert scale with possible responses ranging from ‘not at all’ (1) to ‘very much so’ (5) was used. Cronbach’s alphas were .64 (wealth), .77 (fame), .71 (social concern), .72 (affiliation), .65 (self-growth), and .68 (leisure). Survey items were presented in the Appendix.

### Value placed on learning

Value placed on learning was measured by the question of ‘Things learned in college seemed interesting and useful’. The item was measured with a five-point Likert scale ranging from ‘not at all’ (1) to ‘very much’ (5).

### Intention to change major

Intention to change major was measured by an item of ‘How much of a plan do you have regarding the following activity while in college?: changing my major field of study’ was adopted with a 4-point Likert scale; 1 = ‘none’, 2 = ‘a little’, 3 = ‘somewhat’, and 4 = ‘a lot’. A higher score indicates more intention to change their major.

### Control variables

#### Study time per week

Study time was measured by the question of ‘This year, how many hours per week on average did you spend on the following activities?’. The following options included ‘studying (preview, review, homework, etc.)’ with an 8-point Likert scale; 1 = ‘none’, 2 = ‘less than 1 hour per week’, 3 = ‘1–2 hours per week’, 4 = ‘3–5 hours per week’, 5 = ‘6–10 hours per week’, 6 = ‘11–15 hours per week’, 7 = ‘16–20 hours per week’, and 8 = ‘more than 21 hours per week’.

#### Grade point average (GPA)

A standardized GPA ranging from 0 to 5 was used. Actual GPA score was divided into a full mark (i.e., 4.0, 4.3, 4.5).

#### Self-directed learning ability (SDLA)

A perceived self-directed learning ability was measured by the item ‘How do you consider yourself compared to other people of similar age regarding the following item?: ‘self-directed learning ability’ was adopted. A five-point Likert scale with possible responses ranging from ‘very low’ (1) to ‘very high’ (5) was used.

### Educational and relational satisfaction

Educational and relational satisfaction was measured by the question ‘In the past semester, how satisfied were you with the following components of college life?’. The following options included ‘overall educational context’ and ‘peer relationships’. The item was measured with a five-point Likert scale ranging from ‘very dissatisfied’ (1) to ‘very satisfied’ (5).

### Data analysis

#### Missing data analysis

Across all items, the proportion of missing data ranged between 0 and 0.1% except GPA. GPA had a missing proportion of 1.1%. A missing proportion less than about 5% is likely to be inconsequential for biases and loss of power, allowing for the use of listwise deletion on further analysis.

#### Statistical analysis

Descriptive statistics, including means and standard deviations, were calculated first. We conducted a multivariate analysis of variance (MANOVA) and a post-hoc Bonferroni test to compare mean differences in goal contents among students with different majors. Pillai’s Trace was used when the Box’s Test of Equality of Covariance was statistically significant (*p* < .05). For the first research question, we conducted multiple regression analyses; six goal categories (independent variables), gender (male = 1, female = 2), study time, GPA, and SDLA (control variables) were entered. Multiple regression is a general inferential statistic that describes associations between a continuous dependent or outcome variable and multiple predictors (or independent variables) in one equation. In practice, estimates in multiple regression is preferable for researchers because partial regression coefficient (e.g., β1) suggests strength (i.e., slope) of X1 and dependent variable, controlling for the effect of the others. This controlling may able to infer true relations between the variables (e.g., X1 and Y). Relative contributions of goal contents in explaining the value placed on learning for different major groups were examined. For the second research question, multiple regression analyses were also conducted with six goal categories (independent variables), gender (male = 1, female = 2), educational satisfaction, relational satisfaction, and GPA (control variables) and an outcome variable of intention to change their major. In multiple regression analysis, all *R*^2^ were adjusted, and standardized βs were used to assess the strength of the predictor variables. We set the limit of the variance inflation factor (VIF) of the independent variables to .3, and there was no multicollinearity. We then tested the relationships among the crucial goal content (i.e., social concern goal), value placed on learning and intention to change major with structural equation modeling (SEM) in the AMOS 18 software (SPSS, USA). The root mean square error of approximation (RMSEA), the comparative fit index (CFI) and Tucker-Lewis Index (TLI) were used for assessment of model fit. A Sobel test was used to evaluate the significance of medical school students’ indirect paths from social concern goal through value placed on learning to intention to change major. All statistical tests were two-tailed, with *p* < .05.

## Results

### Descriptive statistics and mean differences among different majors

Table 1 reports descriptive statistics and mean differences by major groups. A MANOVA test, Pillai’s Trace = 0.46, *F* = 5.02, *p* < .001, η_p_^2^ = .02, and follow-up ANOVAs indicated that students with different majors differed in the importance they attributed to different goal categories. Specifically, post-hoc Bonferroni tests indicated that medical school students rated the social concern goal higher and rated the leisure goal lower than engineering students (*p* < .05), but the effect sizes (i.e., eta squares) were small. Affiliation and fame showed no significant differences among groups. The self-growth goal of medical school students was not different from other majors, whereas humanities students emphasized self-growth goal more than than engineering majors.

**Table 1.** Mean differences by major.

Variable/Major	Medicine (*n *= 350)	Engineering (*n *= 418)	Natural Science (*n *= 852)	Humanities (*n *= 318)	*F*(3, 1934)	ηp^2^
Goal categories						
Social concern	3.30(0.06)_a_	3.13(0.60)_b_	3.25(0.59)_a_	3.24(0.62)_a_	8.16***	0.012
Affiliation	3.79(0.55)_a_	3.83(0.58)_a_	3.81(0.57)_a_	3.82(0.57)_a_	0.46^ns^	0.001
Self-growth	3.58(0.52)_ab_	3.58(0.57)_a_	3.66(0.53)_ab_	3.69(0.52)_b_	4.84**	0.007
Leisure	3.26(0.60)_a_	3.38(0.59)_b_	3.36(0.60)_ab_	3.29(0.61)_ab_	4.32**	0.007
Wealth	3.10(0.59)_ab_	3.20(0.65)_a_	3.14(0.58)_a_	2.99(0.68)_b_	9.21***	0.014
Fame	3.69(0.57)_a_	3.69(0.58)_a_	3.77(0.56)_a_	3.67(0.59)_a_	2.27^†^	0.004
Value of learning	3.33(0.78)_a_	3.21(0.82)_a_	3.22(0.87)_a_	3.21(0.85)_a_	1.80^ns^	0.003
Intention to change major	1.54(0.82)_a_	2.08(1.01)_b_	1.99(1.03)_b_	2.07(1.00)_b_	24.43***	0.036
Control variables						
Study time	4.25(1.61)_a_	3.92(1.65)_b_	3.91(1.45)_b_	4.01(1.41)_ab_	3.92**	0.006
GPA	0.74(0.18)_a_	0.71(0.16)_b_	0.73(0.16)_a_	0.74(0.17)_a_	5.31**	0.008
SDLA	3.10(0.82)_a_	3.00(0.85)_a_	3.06(0.88)_a_	3.08(0.86)_a_	1.34^ns^	0.002
Educational satisfaction	3.22(0.85)_a_	3.22(0.86)_a_	3.18(0.91)_a_	3.23(0.86)_a_	0.27^ns^	<0.001
Relational satisfaction	3.92(0.74)_a_	3.91(0.83)_a_	3.81(0.91)_a_	3.83(0.85)_a_	1.95^ns^	<0.001

Standard deviations follow means in parentheses. For the variables, means within a row with non-common subscripts were significantly different using Bonferroni post-hoc tests (*p* < .05).

GPA = standardized Grade Point Average, SDLA = Self-directed Learning Ability.

^†^*p* < .1; **p < .01; ***p < .001. ns = non-significant.

#### Relationships between goal contents and value placed on learning

Table 2 reports the results of multiple regression analyses explaining the variance of value of learning for all major groups. For medical school students, the regression models accounted for 17% of the variance. Controlling for gender, study time per week, GPA and SDLA, social concern goal (β = .22) and self-growth goal (β = .14) showed a significant association with value of learning. A social concern goal was also important in humanities (β = .13) majors, and a self-growth goal was important in engineering (β = .08) and natural science (β = .12) students. An affiliation goal was significant only in natural science (β = .14). In all majors, correlations of leisure, wealth, and fame goals with value of learning were non-significant.

**Table 2.** Multiple regression analyses predicting value of learning.

	Medicine					Engineering					Natural Science					Humanities				
	*b*	*SE*	β	*R^2^*	*F*	*b*	*SE*	β	*R^2^*	*F*	*b*	*SE*	β	*R^2^*	*F*	*b*	*SE*	β	*R^2^*	*F*
Social Concern	0.28	0.08	0.22***	.17	7.23***	0.10	0.05	0.08	.10	10.04***	−0.06	0.08	−0.04	.06	3.84***	0.18	0.08	0.13*	.07	3.82***
Affiliation	0.00	0.09	0.00			0.07	0.06	0.05			0.22	0.08	0.14**			0.03	0.08	0.02		
Self-Growth	0.20	0.10	0.14*			0.12	0.06	0.08*			0.20	0.09	0.12*			0.11	0.09	0.07		
Leisure	−0.03	0.07	−0.03			0.02	0.05	0.02			−0.11	0.07	−0.08			0.01	0.08	0.01		
Wealth	−0.10	0.08	−0.07			0.03	0.05	0.02			0.02	0.08	0.01			−0.08	0.07	−0.06		
Fame	0.04	0.09	0.03			0.09	0.06	0.07			0.05	0.09	0.03			0.13	0.09	0.09		
Control variables																				
Gender	−0.17	0.10	−0.09			−0.13	0.07	−0.07*			0.10	0.09	0.06			−0.28	0.10	−0.16**		
Study time	0.04	0.03	0.08			0.07	0.02	0.15***			0.11	0.03	0.19***			0.09	0.03	0.15**		
GPA	0.60	0.24	0.14*			0.35	0.17	0.07*			0.06	0.28	0.01			0.08	0.28	0.02		
SDLA	0.10	0.05	0.11			0.05	0.03	0.05			−0.03	0.05	−0.03			−0.04	0.05	−0.04		

Gender: male = 1, female = 2, GPA = Grade point average, SDLA = Self-directed learning ability.

*p < .05; **p < .01; ***p < .001.

#### Relationships between goal contents and intention to change one's major

Table 3 reports the results of multiple regression analyses explaining the variance of intention to change one's major for all major groups. For medical school students, the regression models accounted for 6% of the variance. After controlling for gender, educational satisfaction, relational satisfaction, and GPA, the social concern goal showed a negative association (β = −.15) and a wealth goal showed a positive association (β = .12) with intention to change one's major. In contrast to the result of medicine, social concern goal was positively related to intention to change one's major in engineering and humanities students.

**Table 3.** Multiple regression analyses predicting intention to change major.

	Medicine					Engineering					Natural Science					Humanities				
	*b*	*SE*	β	*R^2^*	*F*	*b*	*SE*	β	*R^2^*	*F*	*b*	*SE*	β	*R^2^*	*F*	*b*	*SE*	β	*R^2^*	*F*
Social concern	−0.20	0.09	−0.15*	.06	3.11***	0.15	0.07	0.09*	.06	6.12***	0.03	0.10	0.01	.04	2.71**	0.28	0.09	0.18**	.10	4.84***
Affiliation	−0.13	0.10	−0.08			0.02	0.07	0.01			0.13	0.10	0.07			−0.12	0.10	−0.07		
Self-growth	0.14	0.10	0.09			−0.07	0.07	−0.04			−0.01	0.11	0.00			−0.07	0.11	−0.04		
Leisure	0.09	0.08	0.07			0.05	0.06	0.03			0.06	0.09	0.04			0.00	0.09	0.00		
Wealth	0.17	0.08	0.12*			0.01	0.06	0.01			0.11	0.09	0.06			0.30	0.08	0.20***		
Fame	−0.11	0.10	−0.08			−0.03	0.07	−0.02			−0.09	0.11	−0.05			−0.03	0.10	−0.02		
Control variables																				
Gender	−0.04	0.11	−0.02			0.07	0.08	0.03			−0.20	0.10	−0.10			−0.12	0.11	−0.06		
E_satisfaction	−0.06	0.06	−0.06			−0.13	0.04	−0.11**			−0.14	0.06	−0.12*			−0.25	0.06	−0.21***		
R_satisfaction	−0.06	0.07	−0.05			−0.09	0.04	−0.07*			−0.11	0.06	−0.10			0.03	0.07	0.02		
GPA	−0.62	0.26	−0.13*			−1.14	0.21	−0.19***			−0.56	0.33	−0.08			−0.82	0.32	−0.14*		

Gender: male = 1, female = 2, E_satisfaction = Educational satisfaction, R_satisfaction = Relational satisfaction.

*p < .05; **p < .01; ***p < .001.

#### Relationships among goal contents, value placed on learning and intention to change one's major

**Figure 1.**
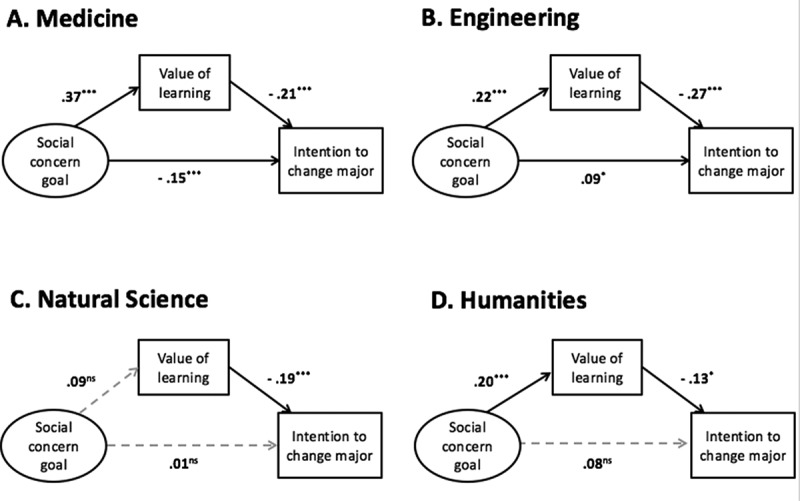
Relationships between social concern goals, value of learning, and intention to change one's major in medical school students. Standardized path coefficients for (a) medicine, (b) engineering, (c) natural science, and (d) humanities are described. Dotted gray line indicates that the coefficient was not significant. *p < .05, ***p < .001. ns = non-significant.

Figure 1 depicts a model illustrating the mediating effect of value placed on learning between a social concern goal and intention to change one's major. This model adequately fits the data in all groups: the RMSEA was .067, the CFI was .970, and TLI was .943 among medical students; the RMSEA was .097, the CFI was .930, and the TLI was .869 among engineering majors; the RMSEA was .057, the CFI was .967, and the TLI was .937 among natural science majors; the RMSEA was .070, the CFI was .959, and the TLI was .924 among humanities students. For medical school students, value placed on learning was a partial mediator (*z* = 8.39, *p* < .001). Social concern goals decreased intention to change a major directly or indirectly by increasing value placed on learning. Being an engineering major also showed significant indirect effect (*z* = 4.64, *p* < .001) on intention to change one's major but the social concern goal directly increased, not decreased, intention to change one's major. There was no mediation effect in natural science majors (*z* = 1.36, *p* < .1), and the mediation in humanities students was equivocal (*z* = 1.94, *p* = .053).

## Discussion

The main aim of the present study was to illuminate the consequences of pursuing different goalss, especially focusing on the social concern goal in medical school students. Our study is the first to investigate the effects of goal contents on medical students’ motivational characteristics. This study demonstrated that what students pursue could predict the value they place on learning and intention to change their major. Even though medical students showed the same level of social concern goal with students of natural science and humanities, the role of social concern was different. Having a social concern goal is crucial to improving medical students’ value of learning and decreasing intention to change their major, after controlling critical variables; gender, study time, GPA, SDLA, and educational and relational satisfaction. As Ryan (1996) says, ‘all goals are not created equal’ [13].

According to the SDT, an intrinsic goal leads to positive effects because the goal is satisfying three psychological needs of autonomy, competence, and relatedness [14,15]. Among the goal contents of this study, having a social concern goal in medical school may lead to the greatest fulfillment of psychological needs. When medical students pursue social concern goals, they may feel personal autonomy and volition, competence, and a significant feeling of connection with other society members. Therefore, developing educational programs or establishing policies to increase medical students’ social concern goal could lead to society’s betterment and individual expertise development.

In line with previous studies, there were overall patterns that a social concern goal, affiliation, and self-growth goals (intrinsic goals) are positively related with value placed on learning, whereas the wealth and fame goals (extrinsic goals) have no significant relationship with them. Previous studies showed that mental health, self-esteem, ecologically adaptive functions, social behavior, and academic performance are rated higher with regard to intrinsic goals [16,17]. Cross-sectional and longitudinal studies revealed that pursuing money-related goals (i.e., extrinsic goals) attracted higher depression, anxiety, and behavioral disorders than pursuing self-acceptance-related goals (i.e., intrinsic goals) [4]. In addition, negative aspects of extrinsic goals were also revealed among Belgian business students [5], American students and adults [18], and Chinese adolescents [19]. These results suggest that the negative aspect of extrinsic goal pursuit persists across cultures.

A social concern goal decreases intention to change one's major, while the opposite holds true in a wealth goal in medicine. Furthermore, a social concern goal decreases the intention to change one's major directly and also indirectly through increased value of learning. Truly, placing value in human-centered treatment rather than a medical doctor’s social status or wealth contributes to improvements in community welfare. This study revealed that having a social concern goal also has a positive effect on the person who pursues that goal. This result provides a reason to encourage development of social concern goals in medical students. Pursuing social concern goals positively predicts behavioral, emotional, and cognitive academic engagement (i.e., elaboration, monitoring, and regulation) in school, after controlling for the mastery and performance goal orientation [20]. Having self-transcendent value helps to increase the mastery approach goal [20], have more meaning in life [21], and an improved well-being [22]. This has implications for developing educational programs that let students know the positive effects of having a social concern goal. The following are possible implements for training health professions: (1) giving medical students opportunities to connect a self-transcendent purpose (i.e., a social concern goal) to self-oriented reasons for medical learning; (2) building social concern motivation by asking open-ended questions about how to increase social justice or social welfare; (3) providing a social norm for medical students by introducing content that shows how ‘medical students like you’ tend to pursue social concern goals, and (4) giving medical students opportunities to write essays that advocate for medical professions students to have social concern goals .

However, a counter-intuitive finding was the positive association between a social concern goal and intention to change their major in engineering and humanities majors. The more students pursue a social concern goal the lower their likelihood to choose engineering or humanities when they have a second opportunity to choose a major. Sophisticated studies are needed to reveal causes of this result. The characteristics of engineering and humanities programs could provide one possible explanation. Engineering is one of the most important fields that contribute to the development of society. However, it is difficult to realize its association during freshman year when one studies mostly basic subjects. In addition, humanities is an academic field that enlightens fundamental questions of value, purpose, and meaning in life using methods of retrospection or criticism [23]. Medicine stresses social contribution and altruism more than engineering and humanities. Even though the questions discussed in engineering and humanities are crucial to long-term improvements in society [24], students could find it difficult to recognize these majors as contributing directly to society. Therefore, a positive association between a social concern goal and intention to change one's major may be observed. This explanation should be tested in future studies.

Based on the perspectives of GCT, social concern, affiliation, and self-growth are categorized as intrinsic goals [25]. However, the roles of specific intrinsic goals were not the same across majors. For instance, social concern goals could help increase both learning motivation and willingness to keep majoring in medical school. These results indicated that the effects of goal contents vary according to the different fields students are involved in, different roles or professions they are working on, and different socio-cultural contexts they belong to. All intrinsic goals do not have the same effects; thus, educators try to define which specific goal students pursue the most and in which context the goal is pursued in reality. The results of this study reinforce the need to prioritize attention to the students’ goal contents early in their studies. Furthermore, in the mean difference analysis, medical students emphasize a social concern goal over a leisure goal in comparison to engineering students. Since the effect sizes were small (.012 for social concern goal, .007 for leisure goal), educators should understand that it is difficult to find differences in goal contents in real practices. The results may reflect the general atmosphere of medical schools that stress hard work and contributing to society. Further studies should examine the underlying mechanisms of these differences.

### Strength and limitations

This study has both strengths and limitations. The data of this study is from a large student population and was collected by a stratifying sampling method in a survey with low missing values (less than 2%). This is the first study to perform quantitative comparisons of six different goal contents among four different majors. Goal research is being performed in the educational field, and numerous articles have addressed the question about the purpose for goal pursuit, such as performance and mastery goals [26,27]. Based on the GCT, this study focused on which contents students pursue in their college life and revealed the most adaptive goal in medical school students. It suggests a path that medical education should pursue. However, since our study is cross-sectional, it does not guarantee causal connections. Even though GCT supports the overall framework of the study, a prospective experimental design would be necessary to draw stronger inferences. Second, the present study used self-reported measures, not focused on the actual behavior. Even though the self-reported survey is an effective way to estimate and quantifying psychological variables, it has become increasingly important to transition from reliance on self-report to reliance on observations of actual behavior [28]. For instance, the results cannot guarantee the actual drop-out rate in medical school, but showed the intention to drop out of the medical school. Therefore, the phrase of ‘intention to change one's major’ was used in the entire text. Further studies should investigate the associations between goal content and actual learning behaviors. Third, the overall variance accounted for in the outcome variables by goal content was modest. Even though researchers suggested that small effects might have larger cumulative effects over time [8], other critical goal contents such as family or appearance should be explored further in future studies.

## Conclusion

In conclusion, this study suggests that in order to enhance medical students’ social concern goal, providing context is necessary in the medical training curriculum, not only for the students’ professional development but also for improving society. Pursuing a social concern goal may both foster the value of learning and reduce the intention to change their major in medical school. This study provides a better, thorough understanding of the goals of medical students and proposes that future studies include these various goal contents.

## Appendix. Survey items

General instructions: This survey is not a test; therefore, there is no right or wrong answer. Please respond to each question honestly according to your own opinion. All survey answers will only be used for the sole purpose of this study and all answers will be kept anonymous. Your answers will contribute greatly to the betterment of our country’s education system.

1 2 3 4 5

not at all somewhat very much so

**Goal contents**

*Social concern*

It is important to give back to society what I gained from society.
I think working for the betterment of society takes up a big part of my life.
I think about how much my future career will contribute to society.
I constantly do community service in order to be of some help to society.

*Affiliation*

I consider it important to form good relationships with other people.
I think forming good relationships with others are more important than any other priorities.
I meet with people around me quite often in order to maintain good relationships.
I tend to contact my friends first in order to maintain our relationships.

*Self-growth*

Continuing one’s effort to develop an individual’s potential is important in life.
Even if I get a high pay rate, a job that doesn’t enable me to develop and grow is unappealing.
If there is a job or activity that can help me develop and grow, I spend time and money very willingly without hesitation.
In my own life, I try to learn from the lives of people who successfully developed and grew in their lives.

*Leisure*

I consider leisure more important than work or study.
A job without sufficient leisure time, even if it brings great wealth, is not a good job.
Even when I have work to do during break, I put leisure first.
I tend to try hard to spend my leisure time to the fullest.

*Wealth*

When making a career choice, annual income is more important than other factors.
I believe wealth is what defines my social status.
For my future, I am interested in investing and managing my finances.
I am interested in stories related to the financial success of big corporation CEOs.

*Fame*

I consider it important to be an authoritative figure in my field of expertise.
I think it is important for other people to recognize me.
I try hard to be someone who others can respect.
I work hard in order to gain social recognition.
